# Implementation of Taguchi and Genetic Algorithm Techniques for Prediction of Optimal Part Dimensions for Polymeric Biocomposites in Fused Deposition Modeling

**DOI:** 10.1155/2022/4541450

**Published:** 2022-01-31

**Authors:** Raman Kumar, Jasgurpreet Singh Chohan, Sandeep Singh, Shubham Sharma, Yadvinder Singh, S. Rajkumar

**Affiliations:** ^1^Department of Mechanical Engineering, Chandigarh University, Gharuan 140413, India; ^2^Department of Civil Engineering, Chandigarh University, Gharuan 140413, India; ^3^Department of Mechanical Engineering, I.K. Gujral Punjab Technical University, Jalandhar-Kapurthala Highway, VPO Ibban 144603, India; ^4^Department of Mechanical Engineering, Faculty of Manufacturing, Institute of Technology, Hawassa University, Awasa, Ethiopia

## Abstract

Additive manufacturing has gained popularity among material scientists, researchers, industries, and end users due to the flexible, low cost, and simple manufacturing process. Among number of techniques, fused deposition modeling (FDM) is the most recognized technology due to easy operation, lower environmental degradation, and portable apparatus. Despite numerous advantages, the limitations of this technique are poor surface finish, dimensional accuracy, and mechanical strength, which must be improved. The present study focuses on the implementation of the genetic algorithm and Taguchi techniques to achieve minimum dimensional variability of FDM parts especially for polymeric biocomposites. The output has been measured using standard testing techniques followed by Taguchi and genetic algorithm analyses. Four response variables were measured and were converted into single variable with combination of different weightages of each response. Maximum weightage was given to width of FDM polymeric biocomposite parts which may play critical role in biomedical and aerospace applications. The advanced optimization and production techniques have yielded promising results which have been validated by advanced algorithms. It was found that layer thickness and orientation angle were significant parameters which influenced the dimensional accuracy whereas best fitness value was 0.377.

## 1. Introduction

**Figure 1:**
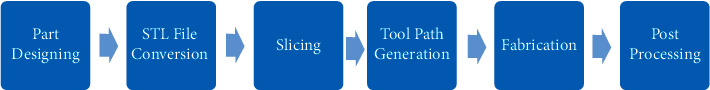
Step-by-step procedure of the FDM process.

Additive manufacturing technologies manufacture the part through layer-by-layer strategy as opposite to conventional subtractive manufacturing techniques [1]. The major advantage of these advanced manufacturing techniques over traditional manufacturing techniques is digitalization of the process which receives input form computer-generated product designs [2–4]. The rapid production and customization of parts with low cost and lower tooling requirements also add to the advantages of these manufacturing strategies [5]. Out of numerous additive manufacturing techniques, fused deposition modeling (FDM) is the most adapted and utilized technique due to lower installation cost and ease of operation [6]. The step-by-step procedure of manufacturing is shown in Figure 1.

**Figure 2:**
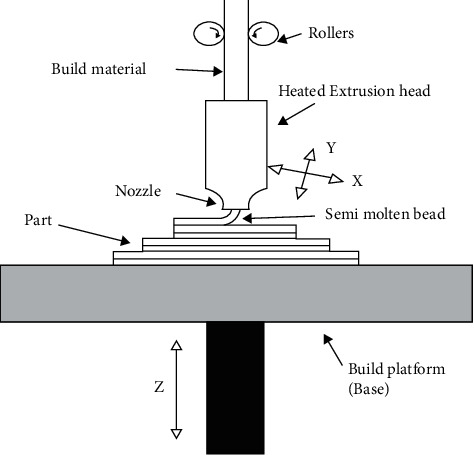
Schematic and components of FDM.

The apparatus of FDM contains extrusion head, nozzle, platform, motors, and microcontroller, which controls the whole operation [7, 8]. The schematic of the FDM process along with major components is shown in Figure 2. As one layer is actually deposited, build platform moves downwards (in *Z* direction), and subsequent level of material is actually deposited, and the process is actually repeated till the desired part is actually attained [9–11]. At times, another filament of washable material is actually utilized to allow for overhanging part that is very easily washed away after fabrication [12].

FFF supplies the personalized products with minimum lead time and manufacturing, cost but the physical strength of part is surely a situation of interest for researchers as extensive variation in physical properties is actually experienced because of perturbation in design [13–15]. Additionally, issues related to lower physical strength of FFF parts might impede the usability of the products for particular programs [16, 17]. Hence, there is surely a necessity of intelligent optimization tools for maximization and prediction of physical strength of FFF parts. There are many input parameters of FFF technology, which have a considerable effect on tensile strength, compressive strength, flexural, and impact strength of FFF part [18–20]. Eventhough many research studies have been carried out for optimization of process parameters of FFF, recent research has focused on development of advanced mathematical tools and hybrid algorithms which may enhance and forecast the physical strength of FFF parts [21–26]. Next section discusses about recent literature on impact of FFF process parameters on mechanical stability and implementation of sophisticated and hybrid algorithms employed for optimization of process parameters of FFF technology [23].

## 2. Literature Review

There are numerous process parameters of FFF technology which have a significant impact of surface quality, mechanical strength, and hardness of fabricated parts. Raster angles of 90° and 0° resulted in higher tensile strength in direction parallel to deposition of filament during the FFF process. On the other hand, positive air gap resulted in smooth surface, which also improves the shore *D* hardness. Gao et al. [24] added polyethylene glycol with different concentrations inside polylactic acid for strength enhancement. It was noticed that bond strength between the layers has significantly increased, whereas mechanical anisotropy was reduced. The intermolecular diffusion and entanglement at bond location were found to be the most possible reason for strength enhancement. In another study [25], the fracture toughness of continuous carbon fiber reinforced nylon composite by varying the printing speed, bed temperature, and nozzle temperature. It was observed that fracture toughness reduces with printing speed increases, whereas an improvement has been noticed with an increase in nozzle and bed temperature. When compared to their traditional equivalents, FFF-fabricated polymer components have weak and anisotropic mechanical characteristics [26].

Many researchers have implemented the advanced optimization tools, artificial intelligence, and machine learning approached. Xue et al. [27] developed a variational autoencoder based upon machine learning and Bayesian optimization for designing a 3D printed prototype with customized macroscopic elastic properties. Goh et al. [28] implemented the neural network technique for exploring the relationship between process parameters and mechanical strength of PolyJet 3D printed parts. Finally, the genetic algorithm was used to identify optimum design conditions to attain desired shore *D* hardness. Another study reported [29] that the hierarchical machine learning was implemented on 3D printed silicone elastomer using freeform reversible embedding, which is difficult due to the need to deposit a Newtonian prepolymer liquid phase within a Bingham plastic support bath. The printed speed was increased more than twice using this optimization tool, whereas mechanical strength was not compromised.

Despite several advantages and potential applications, the major challenge faced by machine learning and artificial intelligence in 3D printing are data acquisition, computational cost, and standards for qualification [30]. Furthermore, in the field of bioprinting, machine learning can be used for optimization of process parameters, minimization of dimensional variability in implants, manufacturing fault detection, and estimation of morphological properties of materials [31].

## 3. Experimentation

### 3.1. Planning of Work

The secondary data have been used for analysis through Taguchi and genetic algorithm processes. Five parameters have been used with three levels each, while four dimensions are measured as given in Table 1. The data of initial and final dimensions of width, length, diameter, and thickness have been used to convert into single response with different weightages.

The maximum weightage of 70% is given to width (*W*), while equal weightage of 10% is given to length (*L*), diameter (*D*), and thickness (*T*). The equation used for conversion is as follows:

(1) $$\begin{array}{c} \text{Mod }W=0.7\Delta W+0.1\Delta L+0.1\Delta T+0.1\Delta \;D. \end{array}$$

## 4. Results and Discussion

The genetic algorithm is a method based on natural selection, the mechanism that drives biological evolution, for addressing both limited and unconstrained optimization problems. A population of individual solutions is repeatedly modified by the genetic algorithm. At each phase, the genetic algorithm chooses parents at random from the current population and utilizes them to generate the following generation's children. The population “evolves” toward an ideal solution over generations. The genetic algorithm may be used to handle a number of optimization problems that are not well suited for traditional optimization techniques, such as issues with discontinuous, nondifferentiable, stochastic, or highly nonlinear objective functions. The evolutionary algorithm can be used to solve issues involving mixed integer programming, in which certain components must be integer-valued. In the present study, the impact of five FFF process parameters, i.e., layer thickness, orientation angle, raster angle, raster thickness, and air gap has been studied on dimensional accuracy of parts. The genetic algorithm approach has been implemented on to calculate Mod W, which is the output of four different dimensional accuracy parameters with different weightages.

**Figure 3:**
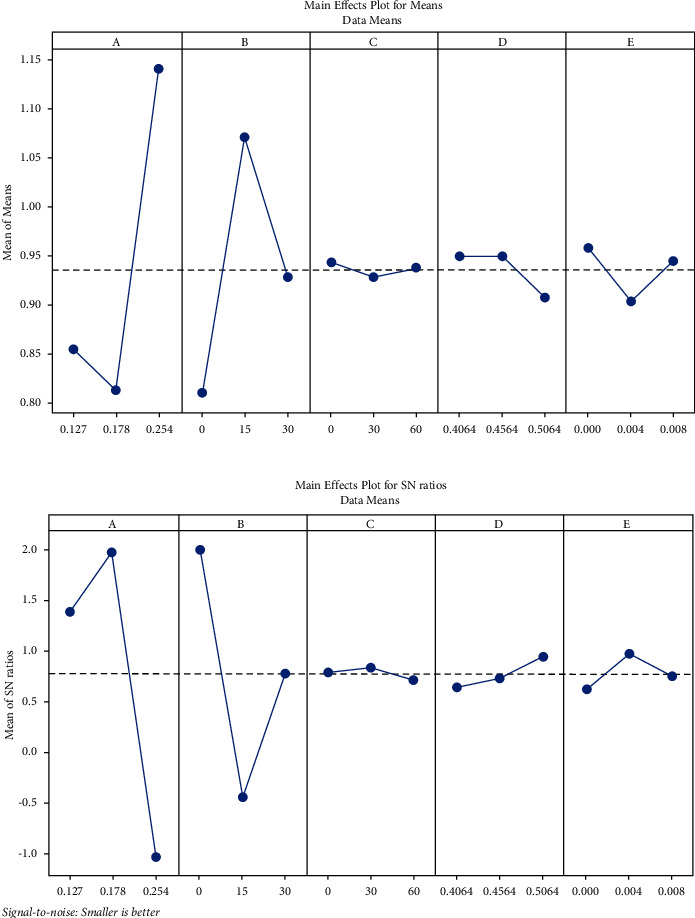
(a) Mean plot. (b) SN ratio plot.

The output in form Mod *W* consists weightages given to different response variables which have been initially evaluated using Taguchi analysis.  Figure 3 shows the mean and SN ratio graphs of output and defines the relationship between input and response. It must be noted that layer thickness is the most prominent parameter followed by orientation angle. The layer thickness of 0.178 mm yielded the maximum value of SN ratio which signifies better dimensional stability. Furthermore, the orientation angle of 0° was optimum for attaining better dimensional accuracy. In case of air gap, the SN ratio is maximum at 0.004 mm, whereas it is reduced by maximum and minimum values of air gap, i.e., 0 mm and 0.008 mm, respectively. The impact of raster angle and raster width is minimum on SN ration of dimensional accuracy. The SN ratio is maximum at 0° and 60° raster angle settings, whereas 0.4064 raster width yielded better dimensional stability.

The significance value and rank of each parameter are given in Table 2, as derived from Taguchi analysis.

The equation has been generated for Mod *W* using regression analysis and described as

(2) $$\begin{array}{c} \text{Mod }W=-3.58+0.0A+0.0096B-0.0064C+20.0\;D-71.4E\\ +40.2A*A-0.000898B*B+0.000012C*C-17.9\;D*D\\ -295E*E-0.0121A*B-0.0302A*C-27.7A*D\\ +155A*E-0.000100B*C+0.0478B*D\\ +1.167B*E+0.0232C*D+0.520C*E. \end{array}$$

It can be observed that parameter *A*, i.e., layer thickness has the maximum impact in dimensional accuracy followed by orientation angle. The analysis using ANOVA has been carried and is given in Table 3. In the present study, the *R*-squared value is 71.28%.

**Figure 4:**
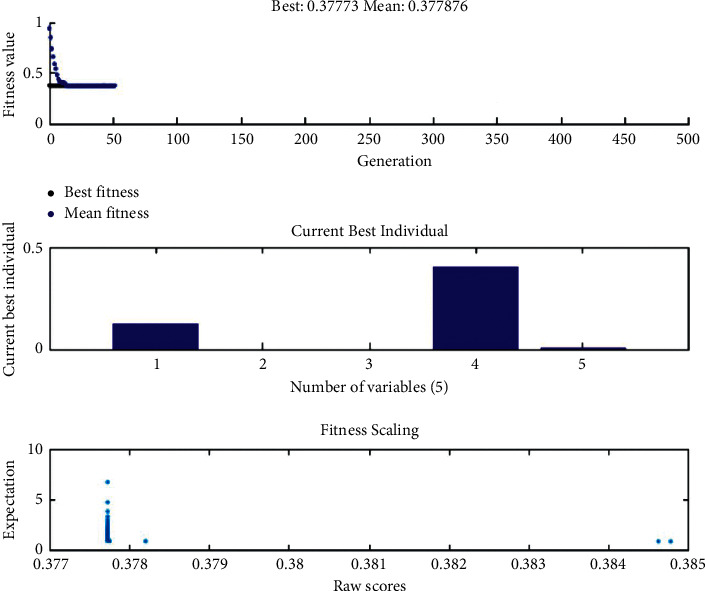
Fitness scaling and best values predicted by the genetic algorithm.

Similar results have been observed after ANOVA analysis which indicates that maximum contribution of 54.06% and 38.35% of layer thickness and orientation angle has been measured. The analysis using the genetic algorithm has been performed, and charts are derived which show the fitness scaling, current best value, and overall best values and means as shown in Figure 4. The charts are plotted between fitness value vs. generation, current best value vs. variable, and expectations vs. raw sores. The results yielded by the genetic algorithm optimized and predicted the results with higher accuracy as compared to conventional optimization techniques. It was predicted that optimum parameter settings would be 0.127, 0, 0, 0.4064, and 0.008 for layer thickness, orientation angle, raster angle, raster width, and air gap, respectively, with objective function value of 0.377730056.

The efficacy of the genetic algorithm is validated as previous studies have found similar results, but time and efforts done to achieve the results were more along with errors in calculations. The parameters used for the genetic algorithm are given in Table 4.

The dimensional accuracy of FDM-printed polymeric biocomposite parts plays the vital role as medical implants are installed in human body, and dimensional variability may cause postsurgery complications [30].

## 5. Conclusions

Fused deposition modeling (FDM) of polymeric biocomposites has been extensively used for rapid tooling, medical implants, aerospace components, engine parts, and household equipment. The process parameters of FDM have significant impact on surface roughness and dimensional variability which must be minimized to increase the usability and applicability of this technique. The dimensional accuracy of FDM-printed polymeric biocomposite parts plays a vital role as medical implants are installed in human body, and dimensional variability may cause postsurgery complications. Also, in some situations, the weightage given in every dimension of part is not same; hence, there is need of the advanced optimization technique which could provide accurate results for complex situations. In the present study, unequal significance was given to response variable, and a combined response factor has been optimized using the genetic algorithm. The results achieved were better as compared to Taguchi analysis and lesser time taken for finding the best value. The predicted results were validated with 99.12% accuracy which indicated the improved efficacy of the genetic algorithm.

## Figures and Tables

**Table 1:** Input and output parameters used for analysis.

Exp. No.	Factors					Responses
	Layer thickness (mm) *A*	Orientation angle (°) *B*	Raster angle (°) *C*	Raster width (mm) *D*	Air gap (mm) *E*	Mod *W* = 0.7Δ*W* + 0.1Δ*L* + 0.1Δ*T* + 0.1Δ*D*
1	0.127	0	0	0.4064	0	0.816457
2	0.127	15	0	0.4564	0.004	0.806392
3	0.127	30	0	0.5064	0.008	0.951515
4	0.127	0	30	0.4564	0.004	0.883827
5	0.127	15	30	0.5064	0.008	0.851524
6	0.127	30	30	0.4064	0	0.686596
7	0.127	0	60	0.5064	0.008	0.791827
8	0.127	15	60	0.4064	0	0.979389
9	0.127	30	60	0.4564	0.004	0.928935
10	0.178	0	0	0.4564	0.008	0.507068
11	0.178	15	0	0.5064	0	1.072302
12	0.178	30	0	0.4064	0.004	0.906102
13	0.178	0	30	0.5064	0	0.731976
14	0.178	15	30	0.4064	0.004	0.899691
15	0.178	30	30	0.4564	0.008	0.819047
16	0.178	0	60	0.4064	0.004	0.66351
17	0.178	15	60	0.4564	0.008	0.999945
18	0.178	30	60	0.5064	0	0.727309
19	0.254	0	0	0.5064	0.004	0.849293
20	0.254	15	0	0.4064	0.008	1.35796
21	0.254	30	0	0.4564	0	1.223476
22	0.254	0	30	0.4064	0.008	1.096522
23	0.254	15	30	0.4564	0	1.433019
24	0.254	30	30	0.5064	0.004	0.962855
25	0.254	0	60	0.4564	0	0.956191
26	0.254	15	60	0.5064	0.004	1.244108
27	0.254	30	60	0.4064	0.008	1.148151

**Table 2:** Response table for means.

Level	*A*	*B*	*C*	*D*	*E*
1	0.8552	0.8107	0.9434	0.9505	0.9585
2	0.8141	1.0716	0.9295	0.9509	0.9050
3	1.1413	0.9282	0.9377	0.9092	0.9471
Delta	0.3272	0.2609	0.0139	0.0417	0.0536
Rank	1	2	5	4	3

**Table 3:** ANONA analysis of response parameters.

Source	DF	Seq SS	Seq MS	*F* value	*P* value	Percentage contribution
*A*	2	56.100	23.0502	12.37	0.001	54.06
*B*	2	36.797	13.3986	7.19	0.006	38.35
*C*	2	0.063	0.0315	0.02	0.983	0.06
*D*	2	0.420	0.2100	0.11	0.894	0.40
*E*	2	0.571	0.2855	0.15	0.859	0.55
Error	16	9.804	1.8627			9.44
Total	26	103.755				

**Table 4:** Genetic algorithm parameters.

Solver	Gamultiobj-multiobjective optimization
Population vector	Double vector
Population size	50
Creation function	Constraint dependent
Crossover function	Intermediate
Scaling function	Tournament

## Data Availability

The data used to support the findings of this study are available from the corresponding author upon request.

## References

[1] Pennington R. C., Hoekstra N. L., Newcomer J. L. (2005). Significant factors in the dimensional accuracy of fused deposition modelling. *Proceedings of the Institution of Mechanical Engineers, Part E: Journal of Process Mechanical Engineering*.

[2] Sahu R. K., Mahapatra S. S., Sood A. K. (2013). A study on dimensional accuracy of fused deposition modeling (FDM) processed parts using fuzzy logic. *Journal for Manufacturing Science & Production*.

[3] Górski F., Kuczko W., Wichniarek R. (2013). Influence of process parameters on dimensional accuracy of parts manufactured using fused deposition modelling technology. *Advances in Science and Technology Research Journal*.

[4] Sudin M. N., Shamsudin S. A., Abdullah M. A. (2016). Effect of part features on dimensional accuracy of FDM model. *APRN Journal of Engineering and Applied Sciences*.

[5] Chohan J. S., Singh R., Boparai K. S., Penna R., Fraternali F. (2017). Dimensional accuracy analysis of coupled fused deposition modeling and vapour smoothing operations for biomedical applications. *Composites Part B: Engineering*.

[6] Alsoufi M. S., Elsayed A. E. (2018). Surface roughness quality and dimensional accuracy—a comprehensive analysis of 100% infill printed parts fabricated by a personal/desktop cost-effective FDM 3D printer. *Materials Sciences and Applications*.

[7] Kumar P., Singh R., Ahuja I. P. S. (2015). Investigations on dimensional accuracy of the components prepared by hybrid investment casting. *Journal of Manufacturing Processes*.

[8] Gurrala P. K., Regalla S. P. (2014). DOE based parametric study of volumetric change of FDM parts. *Procedia Materials Science*.

[9] Kaveh M., Badrossamay M., Foroozmehr E., Hemasian Etefagh A. (2015). Optimization of the printing parameters affecting dimensional accuracy and internal cavity for HIPS material used in fused deposition modeling processes. *Journal of Materials Processing Technology*.

[10] Saqib S., Urbanic J. (2012). An experimental study to determine geometric and dimensional accuracy impact factors for fused deposition modelled parts. *Enabling Manufacturing Competitiveness and Economic Sustainability*.

[11] Singh B., Kumar R., Chohan J. S. (2020). Polymer matrix composites in 3D printing: a state of art review. *Materials Today: Proceedings*.

[12] Papazetis G., Vosniakos G.-C. (2019). Feature-based process parameter variation in continuous paths to improve dimensional accuracy in three-dimensional printing via material extrusion. *Proceedings of the Institution of Mechanical Engineers, Part B: Journal of Engineering Manufacture*.

[13] Tomal A. N. M. A., Saleh T., Khan M. R. (2017). Improvement of dimensional accuracy of 3-D printed parts using an additive/subtractive based hybrid prototyping approach. *IOP Conference Series Materials Science and Engineering*.

[14] Kumar Y. R. (2012). An application of Taguchi’s technique to improve the accuracy of rapid prototyped FDM parts. *International Journal of Materials Engineering Innovation*.

[15] Chohan J. S., Singh R., Boparai K. S. (2019). Effect of process parameters of fused deposition modeling and vapour smoothing on surface properties of ABS replicas for biomedical applications. *Additive Manufacturing of Emerging Materials*.

[16] Chohan J. S., Singh R., Boparai K. S. Multi response optimization and process capability analysis of fused filament fabrication and chemical vapor smoothing operations for rapid casting of biomedical implants.

[17] Kumar R., Singh H. (2018). Exploring the success factors for examining the potential of manufacturing system output. *Benchmarking: An International Journal*.

[18] Singh H., Kumar R. (2013). Hybrid methodology for measuring the utilization of advanced manufacturing technologies using AHP and TOPSIS. *Benchmarking: An International Journal*.

[19] Singh H., Kumar R. (2013). Measuring the utilization index of advanced manufacturing technologies: a case study. *IFAC Proceedings Volumes*.

[20] Chohan J. S., Singh R., Boparai K. S. (2020). Post-processing of ABS replicas with vapour smoothing for investment casting applications. *Proceedings of the National Academy of Sciences, India Section A: Physical Sciences*.

[21] Kumar R., Chohan J. S., Goyal R., Chauhan P. (2020). Impact of process parameters of resistance spot welding on mechanical properties and micro hardness of stainless steel 304 weldments. *International Journal of Structural Integrity*.

[22] Kumar R., Kumar R., Rai J. S., Virk N. S. (2013). Analysis the effects of process parameters in EN24 alloy steel during CNC turning by using MADM. *International Journal of Innovative Research in Science, Engineering and Technology*.

[23] Dixit N. K., Srivastava R., Narain R. (2016). Comparison of two different rapid prototyping system based on dimensional performance using grey relational grade method. *Procedia Technology*.

[24] Gao X., Qi S., Zhang D., Su Y., Wang D. (2020). The role of poly (ethylene glycol) on crystallization, interlayer bond and mechanical performance of polylactide parts fabricated by fused filament fabrication. *Additive Manufacturing*.

[25] Goh G. D., Yeong W. Y. Mode I interlaminar fracture toughness of additively manufactured carbon fibre thermoplastic.

[26] Gao X., Qi S., Kuang X., Su Y., Li J., Wang D. (2021). Fused filament fabrication of polymer materials: a review of interlayer bond. *Additive Manufacturing*.

[27] Xue T., Wallin T. J., Menguc Y., Adriaenssens S., Chiaramonte M. (2020). Machine learning generative models for automatic design of multi-material 3D printed composite solids. *Extreme Mechanics Letters*.

[28] Goh G. D., Sing S. L., Lim Y. F. (2021). Machine learning for 3D printed multi-materials tissue-mimicking anatomical models. *Materials & Design*.

[29] Menon A., Póczos B., Feinberg A. W., Washburn N. R. (2019). Optimization of silicone 3D printing with hierarchical machine learning. *3D Printing and Additive Manufacturing*.

[30] Goh G. D., Sing S. L., Yeong W. Y. (2021). A review on machine learning in 3D printing: applications, potential, and challenges. *Artificial Intelligence Review*.

[31] Yu C., Jiang J. (2020). A perspective on using machine learning in 3D bioprinting. *International Journal of Bioprinting*.

